# Neuroinflammation and MMPs: potential therapeutic targets in neonatal hypoxic-ischemic injury

**DOI:** 10.1186/1742-2094-6-13

**Published:** 2009-04-15

**Authors:** Christopher C Leonardo, Keith R Pennypacker

**Affiliations:** 1 Department of Molecular Pharmacology and Physiology, School of Basic Biomedical Sciences, College of Medicine, University of South Florida, Tampa, FL 33612, USA

## Abstract

Exposure to hypoxic-ischemic insults during the neonatal or perinatal developmental periods produces various forms of pathology. Injuries that occur in response to these events often manifest as severe cognitive and/or motor disturbances over time. Due to difficulties regarding the early diagnosis and treatment of hypoxic-ischemic injury, there is a growing need for effective therapies that can be delivered at delayed time points. Much of the research into mechanisms of neural injury has focused on molecular targets associated with excitotoxicity and free oxygen radicals. Despite repeated success in animal models, these compounds have failed to show efficacy in clinical trials. Increasing evidence indicates that hypoxic-ischemic injury in the neonate is progressive, and the resulting neuropathies are linked to the activation of neuroinflammatory processes that occur in response to the initial wave of cell death. Understanding this latter response, therefore, will be critical in the development of novel therapies to block the progression of the injury. In this review, we summarize emerging concepts from rodent models concerning the regulation of various cytokines, chemokines, and matrix metalloproteinases in response to ischemia, and the various ways in which the delayed neuroinflammatory response may contribute to the progressive nature of neonatal hypoxic-ischemic injury in rat. Finally, we discuss data that supports the potential to target these neuroinflammatory signals at clinically relevant time points.

## Review

### Clinical pathology

Due to improvements in medical care over past decades, increasing numbers of premature and low birth weight infants survive the neonatal period. Not surprisingly, there has been a rise in the incidence of diseases associated with prematurity, including neuropathies. Advances in diagnostic methods for these neuropathies and general knowledge of the cellular and molecular consequences have provided insight into potential causes of these disorders. Hypoxia-ischemia (H-I) is thought to be a major cause of perinatal brain injury, producing lesions of variable severity including focal necrotic cell death, diffuse white-matter injury, and cystic or cavitary infarction. It is estimated that nearly 40% of premature infants may suffer from either intraventricular-periventricular hemorrhage or periventricular lesions. In both cases, age of prematurity and birth weight are associated with increased risk, with younger, lighter infants at highest risk [1].

The escalating incidence of these deficiencies and lack of effective therapies underscores the importance of research in this area. Rodent H-I models have provided strong and convincing evidence supporting the detrimental roles of oxidative stress and free radicals, blood-brain barrier (BBB) disruption, cytokine and chemokine signaling, and matrix metalloproteinase (MMP) activity after H-I. In fact, there is growing evidence demonstrating a discreet temporal injury profile whereby neural injury is exacerbated due to neuroinflammatory signaling from activated microglia and peripheral macrophages. This review highlights the roles of inflammatory cytokines, chemokines, and MMPs after ischemia, and the precise manner in which these proteins may mediate perinatal H-I injury in rat brain.

### Energy failure and free radicals: the early response

In the developing brain, H-I results in a biphasic injury profile. Many of the initial events leading to progressive damage have been identified. The ischemic brain relies heavily on anaerobic glycolysis, which is energetically unfavorable [2]. Energy failure initiates a series of deleterious biochemical cascades. Free oxygen radicals produced from xanthine byproducts and prostaglandin synthesis attack polyunsaturated fatty acids of the plasma membrane, increasing membrane permeability. The mitochondrial respiratory chain is also a major source of reactive oxygen species (ROS), and mitochondrial dysfunction contributes to cellular necrosis as well. Endothelial cell death compromises the BBB, resulting in vasogenic edema and hemorrhage [3]. The failure of energy-dependent ion pumps causes cellular depolarization and glutamate release into the extracellular space. NMDA receptor activation increases intracellular calcium, contributing to the production of ROS and nitric oxide (NO). Lipid peroxidation occurs when NO combines with superoxide to form peroxynitrite [4].

Thus, the early response to H-I is characterized, in large part, by neuronal necrosis resulting from excitotoxic damage. While much of this injury is irreversible, the progressive nature of this pathology offers potential for intervention at delayed time points when neuroinflammatory mediators modulate the neural response to insult.

### Neuroinflammation: the delayed response

The contributions of excitotoxicity and free radical production to the early ischemic response are clear, yet the selective targeting of these mechanisms has not led to significant functional improvements in clinical trials. Importantly, a second wave of cell death occurs in response to ischemic insult and is mediated by the neuroinflammatory response to injury. Accumulating evidence suggests that targeting delayed neuroinflammatory mechanisms may be a promising avenue for therapeutic intervention.

The immune response in brain is complex and highly regulated by a host of different cell types and signaling pathways. Resident microglia are among the first to become activated [5]. These cells migrate to necrotic regions where they remove cellular debris from the interstitial space. Also during this time, activated astrocytes upregulate glial fibrillary acidic protein and migrate to the injured site, a phenomenon known as reactive astrogliosis. Cells in and around the lesion core upregulate the expression of lecticans, and these proteoglycans are deposited into the extracellular space [6]. Thus, a dense composition of proteoglycans, reactive astrocytes and microglia invade the injured site and form a tissue barrier referred to as a "glial scar" [7]. The formation and progression of glial scarring is thought to be an innate protective mechanism in brain to isolate the injured area from viable surrounding tissue. As time progresses, however, the glial scar prevents neuroplasticity and repair at the lesion site while activated astrocytes and microglia promote further injury by secreting proinflammatory cytokines and chemokines, thus attracting peripheral macrophages [8]. In concert, these immune cells and proinflammatory molecules perpetuate a feed-forward inflammatory response by further enhancing microglia and macrophage recruitment to the injured site [9]. The end result is a heightened state of inflammation in the brain that promotes cell death.

Cytokines seated on cell surfaces are activated and subsequently released into the extracellular milieu, where they serve as important mediators of apoptotic cell death. Following ischemic insult, activated microglia upregulate the expression of various cytokines and chemokines [10-12]. In particular, tumor necrosis factor alpha (TNF-α) and interleukin-1 beta (IL-1β) have been shown to potentiate the neuroinflammatory response after H-I [13-15]. Interleukin-1 type 1 receptor (IL-1R1) knockout mice showed significant reductions in the expression of macrophage inflammatory protein-1 alpha (MIP-1α), MIP-1β, monocyte chemoattractant protein (MCP) and RANTES (regulated upon activation, normal T cell expressed and secreted) both 18 hrs and 72 hrs after H-I compared to non-transgenic controls. These effects were also associated with reduced leukocyte infiltration [16], suggesting that IL-1β mediates chemotaxis of peripheral immune cells to the injured site. Recent data combining *in vitro *and *in vivo *methodologies also suggests that MCP-1 is a key mediator of the microglial chemotactic response to neonatal hypoxia. In these experiments, hypoxia resulted in a robust increase in activated microglia in the periventricular white matter, a region of selective vulnerability. Additionally, intracranial injection of MCP-1 induced a robust migratory response of CD11b-positive microglia, and hypoxic primary microglial cultures from rat neonates showed increased expression of MCP-1 [17]. Other data from acute rat hippocampal slices showed that activation of alpha chemokine receptor 4 (CXCR4) by the natural ligand stromal cell-derived factor-1 (SDF-1) caused glutamate to be released from astrocytes. Interestingly, this response was found to be dependent on the release of TNF-α following CXCR4 activation, and was amplified in the presence of lipopolysaccharide-stimulated microglia [18]. These results lend further support to the notion that microglia, working in concert with proinflammatory cytokines and chemokines, promote injury after insult.

In contrast to these findings, evidence supporting a neuroprotective role of microglial activation and cytokine signaling has raised issues as to the precise role of this signaling in various injury models. For example, experiments using primary cultures from rat showed that while cultured microglia upregulated TNF-α and IL-1β only in response to mild hypoxia-induced neuronal death, these same activated microglia were protective when added to separately cultured neurons. Furthermore, microglia co-cultured with media from severely compromised neurons released neurotrophic factors [19]. Similarly, other data exists demonstrating that TNF-α is mainly produced by resident microglia and peripheral leukocytes, and TNF-α knock-out mice displayed reduced infarction following permanent middle cerebral artery occlusion (MCAO) [20]. Therefore, because microglial-derived cytokine signaling may serve as either proinflammatory or neuroprotective, the overall effects on a given system are dependant upon a host of factors including the experimental model, the specific state of the microenvironment, the severity of the injury, and the temporal inflammatory profile.

### Matrix metalloproteinases and blood-brain barrier degradation

Although the precise mechanisms have yet to be elucidated, data from numerous studies suggests that MMPs are instrumental in the production and maintenance of a proinflammatory microenvironment [21]. Collectively, MMPs are capable of proteolytically cleaving all extracellular matrix (ECM) proteins [22]. In addition to ECM degradation, however, MMP activity is a well-known contributor to ischemic neuropathology.

In the developing brain, microglia mediate the neuroinflammatory response through a variety of mechanisms [8]. In the case of ischemia, activated microglia upregulate the expression of cytokines and chemokines, enhancing the inflammatory response [10-12]. Increased expression of MMPs, particularly gelatinases, occurs concomitantly. The release of proteases from activated microglia results in proteolytic degradation of basement membrane constituents [23]. Extravasation resulting from BBB degradation permits entry of peripheral monocytes and macrophages into the brain, further enhancing the recruitment of immune cells to the lesion site [9]. Infiltrating leukocytes [24] are also sources of MMPs, though there is some evidence that microglia are the primary macrophages that respond to neonatal ischemic injury [25].

*In vivo *experiments revealed MMP-9 expression that was localized to neutrophils and endothelial cells, and showed elevated MMP-2 expression in astrocytic endfeet [26]. The latter expression profile places MMP-2 in the ideal position to proteolytically process basement membrane proteins, as astrocytic endfeet play an important role in sealing the BBB. An elegant study later supported this mechanism by showing that laminin is a key substrate for MMPs after ischemia. Gelatinase activity increased in cortex after MCAO and active MMP-9 was upregulated and proteolytically processed laminin when incubated with tissue homogenates from ischemic brain. These effects were attenuated after administration of a highly selective gelatinase inhibitor [27]. There is now substantial data demonstrating the degradation of basement membrane proteins by several MMPs that are elevated after ischemia [3,21,28].

Though the relative contributions of the resident and peripheral immune responses have not been elucidated, a recent study showed that resident microglia were associated with neural injury resulting from oxygen glucose deprivation in organotypic hippocampal slices from rat neonates. In these experiments, CD11b – positive microglia expressed MMP-9 after insult, and increased microglial MMP-9 expression occurred concomitantly with enhanced neurodegeneration and gelatinolytic activity [29]. Thus, in the absence of the peripheral immune response, resident cells of the brain were sufficient to exacerbate injury that was associated with microglial activation and MMP activity. Future studies are needed to delineate the relative contributions of resident microglia and peripheral macrophages *in vivo*.

### Sheddase activity and extracellular matrix remodeling

Accumulating evidence suggests that gelatinases may contribute to the inflammatory response through "sheddase" activity, the process by which MMPs cleave proinflammatory cytokines attached to cell surfaces. This proteolytic processing activates and releases these molecules into the extracellular milieu to exert a wide range of effects depending upon the specific state of the microenvironment. MMPs have previously been shown to process both TNF-α [30] and IL-1β [31] to their biologically active forms. In culture, MMP-2 – positive astrocytes produced MMP-9 when stimulated with either TNF-α or IL-1β [32]. In agreement with these data, mice lacking MMP-9 showed improved outcomes that were directly related to reduced microglial activation [33], attenuated BBB degradation [24] and limited white matter damage [34]. Other data showed that gelatinases cleaved SDF-1α and various MCP chemokines [35], and SDF-1 accelerated proteolytic processing of syndecans by MMP-9 [36]. Interestingly, a recent study performed in the rat neonate showed that lectin-positive microglia/macrophages upregulated syndecan-2 expression in response to hypoxia, and exogenous application to hypoxic primary microglial cultures resulted in increased production of TNF-α, IL-1β and ROS [37]. Cerebral blood vessels also express high levels of syndecans, which modulate transendothelial migration of monocytes across the brain endothelium [38]. Taken together, these data offer potential mechanisms by which gelatinase activity enhances neuroinflammation after H-I by facilitating immune cell chemotaxis.

In addition to their roles in activating cytokines and processing basement membrane proteins, MMPs are efficient at cleaving lecticans [39]. Lectican proteoglycans are upregulated in glial scars [6], where their growth-inhibitory properties maintain a barrier between injured and viable tissue [7,40]. Thus, lectican proteolysis by MMPs may have profound effects on perinatal brain plasticity after injury. Both full-length brevican and the G1 proteolytic fragment were reduced 1 and 14 days after insult in hippocampi of neonatal rats that were exposed to H-I [41]. While the mechanisms of this loss were unclear, the temporal association of brevican loss with lesion progression suggests that brevican expression may be critical for cell viability in the developing brain. In this context, brevican loss resulting from either proteolytic cleavage or cellular injury may enhance neural cell death. Though MMPs are upregulated in glial scar after spinal cord transection [42], very little is known regarding the specific effects of MMPs on glial scar formation and ECM proteolysis after perinatal H-I. Potential mechanisms may include the induction of cellular anoikis or the facilitation of microglia and macrophage migration via reduced steric hindrance resulting from increased ECM clearance.

### Growth factor substrates for matrix metalloproteinases

Although MMP activation is often associated with proinflammatory processes, data from numerous studies demonstrates the ability of MMPs to alter cell growth, signaling and migration in a neuroprotective fashion [43]. For example, MMPs have been shown to cleave ECM proteoglycans that are associated with growth factors, thus releasing these growth factors from sequestration. This has been demonstrated for fibroblast growth factor (FGF) in human endothelial cells after cleavage of the heparin sulfate proteoglycan perlecan [44], as well as for transforming growth factor beta via decorin proteolysis [45]. Similarly, MMP cleavage of non-matrix proteins such as insulin-like growth factor binding protein 3 (IGFBP-3) can activate IGF in dermal fibroblast cultures [46]. Consistent with these data, blocking MMP activity with an endogenous inhibitor increased IGFBP-3 expression and reduced IGF-1 receptor signaling in a murine hepatic tumor model [47].

Other evidence suggests that gelatinase MMPs can act on growth factor receptors to alter receptor turnover. In experiments that used a cervical carcinoma cell/mixed lymphocyte co-culture system to study the effects of immunosuppression, MMP-9 was shown to cleave interleukin-2 receptor alpha on T-lymphocytes [48]. Additionally, MMP-2, but not MMP-9, cleaved FGFR1 from the cell surface, releasing a soluble form into the ECM that retained FGF-binding activity [49]. Other known substrates for gelatinases include vascular endothelial growth factor and MCP-3, the cleavage of which could affect chemotaxis and thus alter the inflammatory response [50,51]. Although there is a paucity of data from *in vivo *ischemic models linking MMP activity to growth factors, the aforementioned studies illustrate the distinct ways in which MMPs modulate numerous cellular processes and may, in fact, provide protection depending upon the temporal activation profile and the overall conditions of the microenvironment.

### The therapeutic window: can we neuroprotect at delayed time points?

To date, the majority of research into neonatal H-I has emphasized the contributions of glutamate excitotoxicity and free radical production to the resulting neuropathology [4]. Consistent with evidence supporting the roles of free radical production and oxidative stress [52-55], therapeutic approaches targeting these mechanisms in rodents have been of some benefit [56,57]. Nonetheless, most studies showing improved outcomes have limited clinical relevance as protective agents were administered prior to or shortly after insult. Thus, the search continues for more selective therapeutics that do not interfere with critical cellular functions [58] and can be administered at clinically relevant time points.

Data from previous experiments lends support to the notion that selective targeting of MMP-2 and MMP-9 should be considered in developing novel therapeutics. MMP-9 knockout mice showed improved outcomes after cerebral ischemia using either the Vannucci model that mimics neonatal H-I [33,59] or the MCAO model that mimics adult stroke [24,34,60]. AG3340 (prinomastat), a small molecule hydroxamate-based inhibitor of MMPs, is a potent inhibitor with high nanomolar affinity for gelatin-degrading MMPs. The effects of gelatinase inhibition were examined in a model of chronic cerebral hypoperfusion, where data revealed that AG3340 provided neuroprotection in adult rats and mice when administered just prior to insult. Importantly, reduced activation of astrocytes and microglia was associated with retained BBB integrity [61]. In a mouse model of MCAO, the gelatinase-selective compound SB-3CT reduced infarct volume when administered either 2 or 6 hrs, but not 10 hrs, after insult [27].

Minocycline is another compound that has shown great promise as a therapeutic to combat neonatal H-I. Neuroprotective effects have been shown previously [62,63], yet the question remained as to whether efficacy could be achieved at clinically relevant time points. The therapeutic window was subsequently extended when administration 2 hours after H-I resulted in preservation of white matter that was associated with reduced microglial activation [64]. Although minocycline exhibits anti-inflammatory properties, it has also been shown to exert MMP inhibition both *in vitro *and *in vivo *[65]. Indeed, another recent study confirmed the efficacy of both minocycline and AG3340 in reducing microglial activation, reactive astrogliosis and neurodegeneration in the rat neonate when administered 24 hours after H-I [66], further demonstrating not only that MMP activity is linked to neuroinflammation, but also that targeting each of these mechanisms is efficacious at delayed time points and therefore may prove efficacious in the clinical setting.

Despite these encouraging data, it will be critical to proceed with caution when developing novel therapeutics. A hallmark of the neonatal brain is the unique potential for plasticity. Although advantageous from a treatment perspective, it is also likely that administration of exogenous compounds could alter critical developmental processes and/or mechanisms associated with neuroplasticity. It is now known that neurogenesis is prominent after ischemic insult in the neonatal rat brain. The Levison laboratory found that neural progenitors from the subventricular zone (SVZ), and possibly glial progenitors, migrate to and populate columnar regions of the neocortex that show prominent cell death in response to H-I [67]. Another study in mouse showed that treatment with the broad-spectrum MMP inhibitor GM6001 once daily for 10 days inhibited the migration of neural progenitors from the SVZ to the corpus striatum after transient MCAO [68]. Although it is presently unknown whether the migration of neural progenitors results in synapse formation that restores function, prolonged MMP inhibition could prohibit proteolytic cleavage of ECM proteins that is necessary for migration of progenitors, subsequent neuroplasticity and repair. Similarly, inhibition of MMPs could reduce chemokine signaling through reduced processing of MCP chemokines and SDF-1α, thereby reducing progenitor cell migration. Additional concerns need to be considered regarding the inhibition of MMPs, particularly MMP-9, and the potential effects on myelination during a critical period of oligodendrocyte proliferation and maturation. Nonetheless, experimental research has provided strong and convincing evidence that MMP inhibitors are excellent candidate therapeutics if administered selectively at the appropriate time points.

## Conclusion

**Figure 1:**
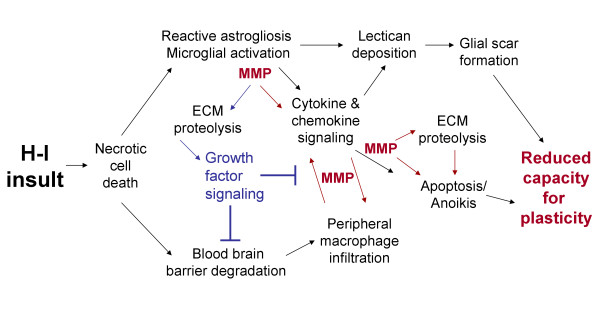
**MMP activity is linked to neuroinflammation and injury progression**. Necrotic cell death after H-I leads to BBB degradation, reactive astrogliosis and activation of resident microglia. Lectican deposition contributes to glial scar formation. Immune cells of the brain increase expression and secretion of proinflammatory cytokines and chemokines. Gelatinase activity initiates a second, delayed opening of the BBB through proteolytic processing of basement membrane constituents. Peripheral macrophages infiltrate into the brain and further promote the inflammatory response. Ultimately, these processes create unfavorable conditions for neuroplasticity and repair. While gelatinases activate cytokines and chemokines through sheddase activity, they also proteolytically process ECM to release growth factors from sequestration. Red arrows indicate neurodegenerative effects; blue arrows indicate neuroprotective effects.

Neonatal hypoxic-ischemic injury is progressive and leads to debilitating neuropathies later in development. Difficulties in diagnosis and treatment underscore the need for novel therapeutics that can be administered at delayed time points. The initial injury results from energy failure and cytotoxicity, yet the delayed neuroinflammatory response is a more viable option for treatment in the clinical setting. As accumulating evidence from rodent models suggests that gelatin-degrading MMPs mediate the neuroinflammatory response to H-I in the developing brain, detailed investigations into the concerted actions of proinflammatory cytokines, chemokines, and MMPs (Figure 1) may prove beneficial in developing novel therapies to treat H-I injury. Care must be taken to ensure that novel therapeutics do not interfere with critical developmental processes and/or limit the potential for neural plasticity.

## Competing interests

The authors declare that they have no competing interests.

## Authors' contributions

CL drafted and edited the manuscript. KP assisted in the editing of the manuscript. All authors read and approved the final manuscript.
